# In the shadow of a megalopolis, a new *Flexamia* from a threatened grass species in the New Jersey Pine Barrens (Hemiptera, Cicadellidae, Deltocephalinae, Paralimnini)

**DOI:** 10.3897/zookeys.511.9572

**Published:** 2015-07-02

**Authors:** Andrew Hicks

**Affiliations:** 1 University of Colorado, Museum of Natural History, Entomology Section, Boulder, CO USA

**Keywords:** Hemiptera, Cicadellidae, Deltocephalinae, leafhopper, *Flexamia*, new species, *Muhlenbergia torreyana*, New Jersey Pine Barrens

## Abstract

A previously unknown species of the North American leafhopper genus *Flexamia*, *Flexamia whitcombi* **sp. n.**, is described from pinebarren smokegrass (*Muhlenbergia torreyana* (Schult.) Hitchc.), a state-listed threatened grass in the New Jersey Pine Barrens. The *serrata* species group, to which it belongs, is redefined and a key to the species of the group is provided. This is the first reported insect association with *Muhlenbergia torreyana*.

## Introduction

*Flexamia* is a charismatic North American leafhopper genus of specialist grass feeders with the species typically feeding upon a single, widespread host species. The hosts they spend their lives on include some of the iconic grasses of the American prairie, rangelands, and deserts, and *Flexamia* are often described as prairie or grassland leafhoppers. By these standards, the new species described herein is an outlier, occurring in the most densely populated state in the US where its host *Muhlenbergia torreyana* (Schult.) Hitchc. grows, though neither New Jersey nor the Pine Barrens—also known as the New Jersey Pinelands—are usually thought of in a grassland context.

**Figure 1.**
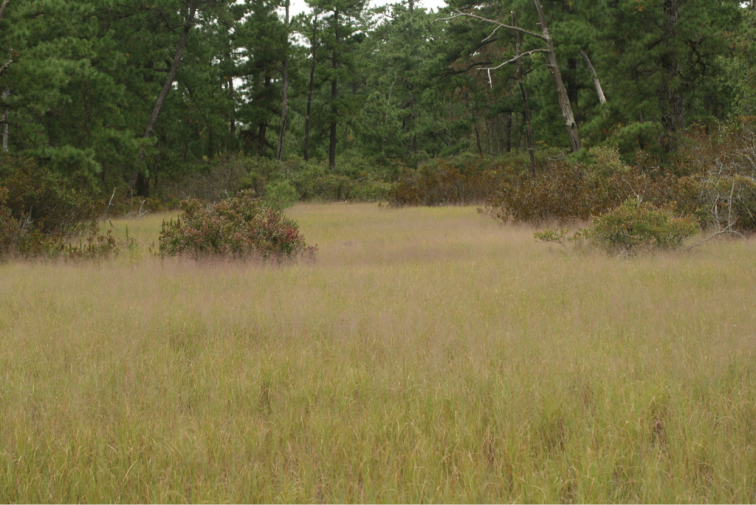
A stand of *Muhlenbergia torreyana* in the Pine Barrens. Photo courtesy of Uli Lorimer of the Brooklyn Botanic Garden.

Known colloquially as pinebarren smokegrass, Torrey’s dropseed, Torrey’s muhly, and New Jersey muhly, *Muhlenbergia torreyana* is a native perennial C4 Chloridoid grass, considered either to be a southern coastal plains species at or near the northern edge of its range in New Jersey (Caiazza and Fairbrothers 1980; McAvoy and Wilson 2014), or a species with centers of distribution in both the New Jersey Pine Barrens and the coastal plain of the Carolinas (Sorrie et al. 1997). *Muhlenbergia torreyana* is rare further south (ibid), with only 13 populations in North Carolina (North Carolina Natural Heritage Program 2015) and “a few” occurrences in Tennessee (NatureServe 2015). Apparently extirpated in parts of its former range including New York (Zaremba and LaMont 1993), *Muhlenbergia torreyana* is locally abundant in wet meadows and seasonally flooded depressions in the Pine Barrens (Fig. 1) and its status is listed as threatened in New Jersey (Special Plants of New Jersey 2013). This is the first report of a leafhopper—or any other insect—using this plant as a host and represents a departure for a member of the species group *serrata* which were previously known only from *Muhlenbergia richardsonis* (Trin.) Rydb. (Whitcomb and Hicks 1988, Bess and Hamilton 1999).

Because of the similarity of its specific epithet, it is also occasionally confused with *Muhlenbergia torreyi* (Kunth) Hitchc. ex Bush, a species known as ring muhly from the southwestern USA.

## Materials and methods

Thirty-five specimens (excluding juveniles) belonging to the new taxon were collected on the host plants by sweeping in two localities. Dissection, measurements, and photographs were completed with the use of a stereo microscope with a digital camera attachment. Genitalia were prepared following techniques found in Oman (1949) and Young and Beirne (1958). Photographs were processed with Helicon Focus photo-stacking software and post-processed using the 5th generation of the standard image-editing software. Micrographs of the aedeagus were acquired using a JEOL JSM-6480 Scanning Electron Microscope.

## Systematics

The *serrata* species group of the genus *Flexamia* was erected by Whitcomb and Hicks (1988) for *Flexamia serrata* Beamer & Tuthill based on the morphology the aedeagal apex, specifically the presence of the pair of dorsal processes. To accommodate *Flexamia huroni* Bess & Hamilton, in which the paired processes were described as apicolateral or lateral terminal, not dorsal, the *serrata* group is redefined here as having 5 or more unbranched processes on the aedeagal apex.

### Flexamia whitcombi sp. n.

Taxon classificationAnimaliaHemipteraCicadellidae

http://zoobank.org/10051907-42E6-4AB7-BFD1-7808B5C701AA

#### Description.

**Figure 2.**
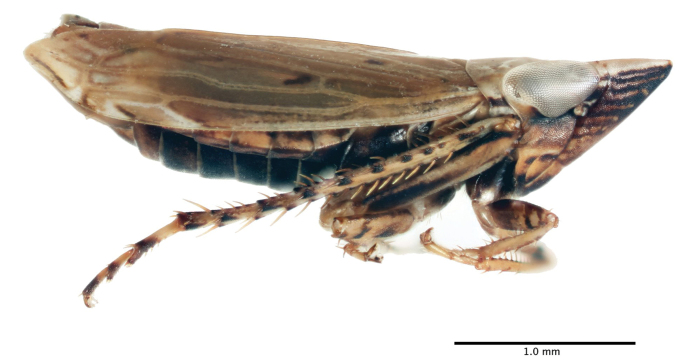
Habitus, male, lateral aspect.

**Figure 3.**
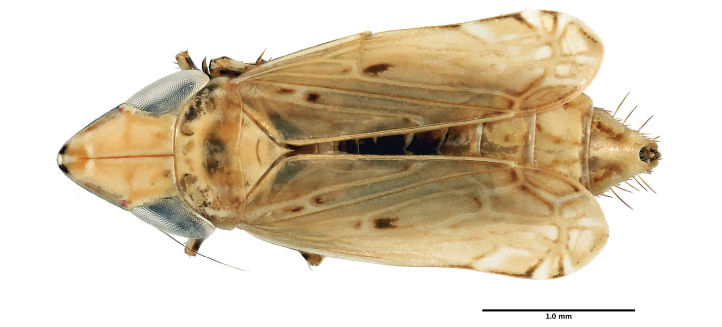
Habitus, female, dorsal aspect.

Length of male 3.5–3.8 mm, length of female 3.7–4.2 mm; head with length of crown ca. 1.5 times interocular width and ca. 0.68 times transocular width. Base color of dried specimens (Figs 2 & 3) usually stramineous, occasionally ivory above, venter entirely or partially fuscous often lighter caudally, occasionally merely stramineous; crown and pronotum without well-defined fuscous spots or stripes except for pair of dark spots at crown apex. Fore-wing typical of the genus, a few scattered small irregular fuscous markings present, veins slightly paler, apex of the abdomen usually exposed on female specimens.

**Figure 4.**
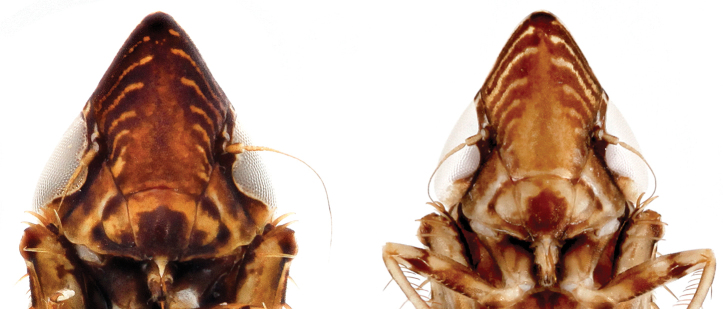
Face, ventral aspect, showing variation in amount of pigmentation.

Face (Fig. 4) coloration varies but typically heavily pigmented apically and laterally, paler medially, 5–6 fine pale transverse lines between eyes not meeting medially, apex of clypellus dark.

**Figure 5.**
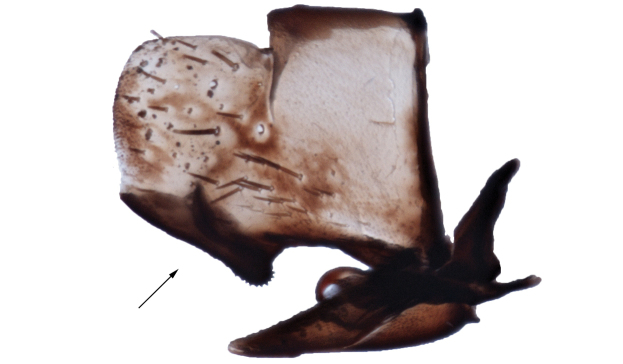
Pygofer and subgenital plates, male, lateral aspect. Note heavily sclerotized caudoventral margin (arrow) and length of subgenital plates relative to pygofer.

**Figure 6.**
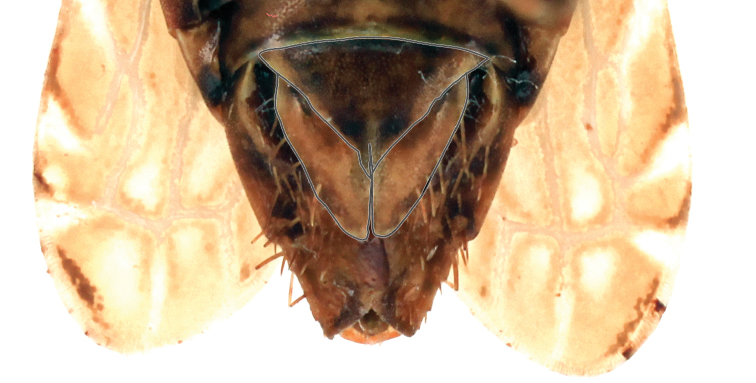
Subgenital plates, ventral aspect, outlined for clarity.

**Figure 7.**
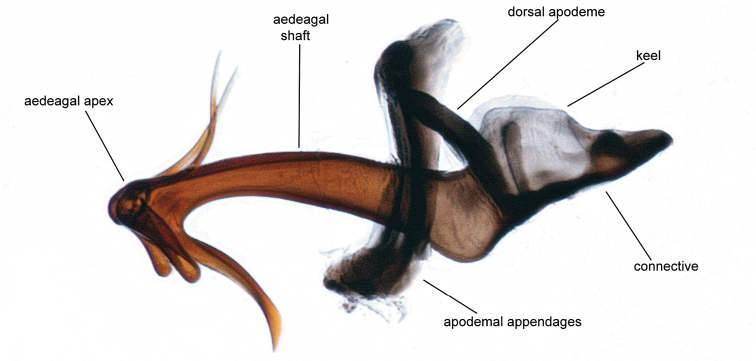
Aedeagus, connective, lateral aspect.

**Figure 8.**
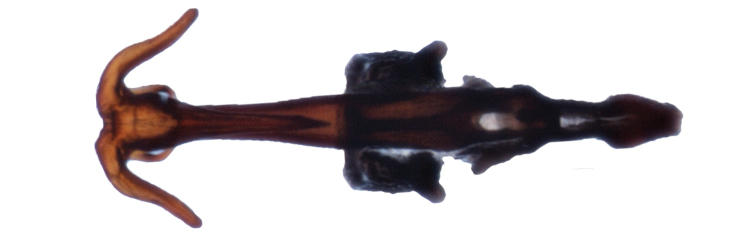
Aedeagus, connective, ventral aspect.

**Figure 9.**
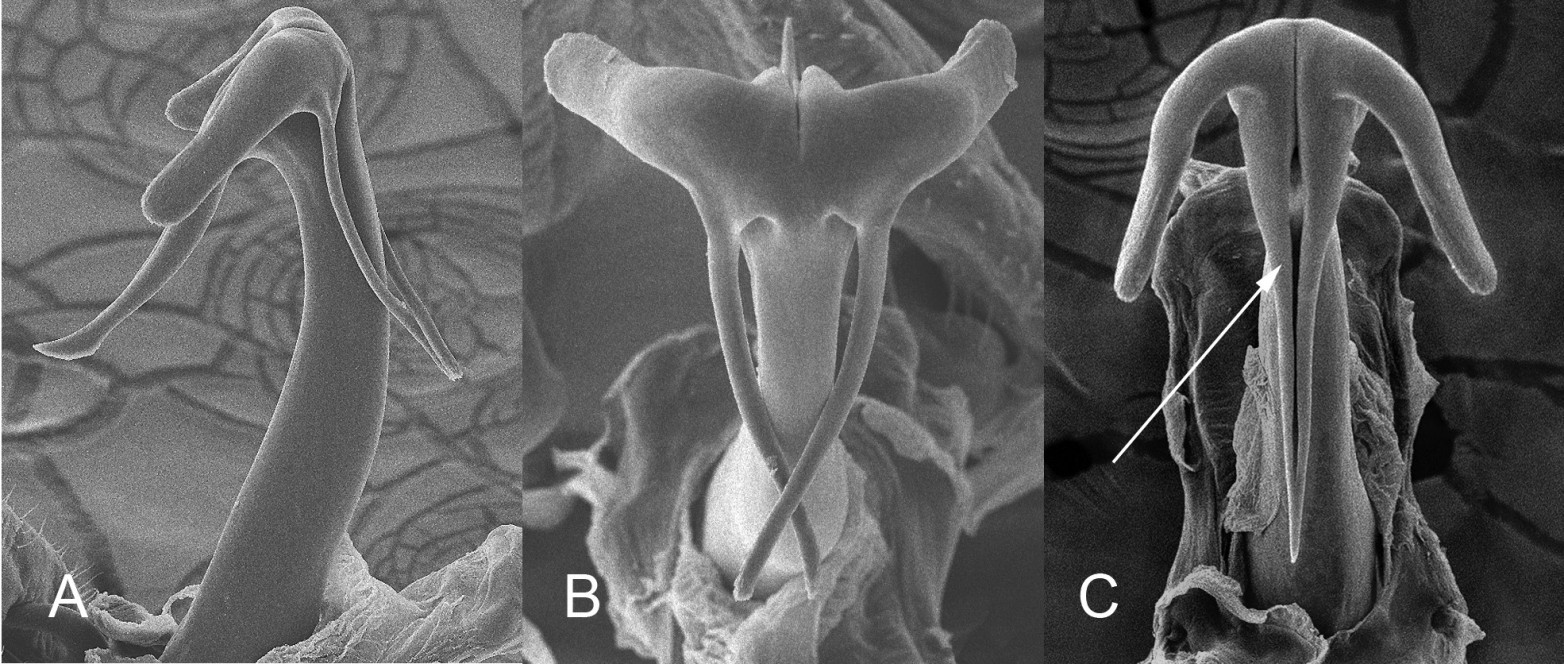
SEMs of the aedeagal apex, from left to right, lateral, dorsal, and caudoventral aspects, the latter illustrating the position of the gonopore on the ventral unpaired process.

Male genitalia. Pygofer (Fig. 5), with posterior lobe truncate apically, caudoventral margin heavily sclerotized, angled and terminating ventrally in a rounded process bearing fine denticulation. Subgenital plates short, extending about 2/3 length of pygofer (Fig. 6), apices relatively blunt, rounded. Connective fused to the aedeagus, keel extending dorsad slightly less than half the height of the dorsal apodeme (Fig. 7). Styles typical of the genus. Aedeagus symetrical, straight in ventral aspect (Fig. 8); in lateral aspect (Fig. 7) shaft long, recurved, tapering evenly before expanding apically, apex (Fig. 9) with 5 processes: on the caudoventral surface, extending laterally and curved ventrad, one pair of short, stout divergent processes with blunt apices, also on the caudoventral surface a longer acute unpaired process extending basad, curved ventrad and bearing the gonopore (Fig. 9C) in the form of a slit extending from the apex of the aedeagal shaft ending subapically on the unpaired process; on the dorsal surface a pair of long spine-like processes which usually cross over the shaft of the aedeagus (Fig. 9A, B). Both apex of shaft and pygofer process occasionally visible in undissected specimens.

**Figure 10.**
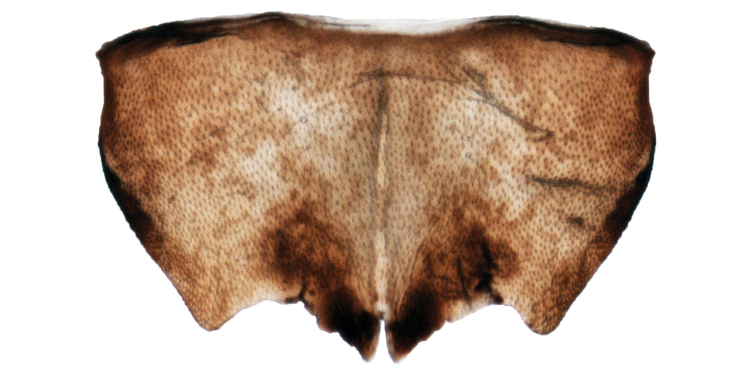
Female 7^th^ sternite. Commonly illustrated for this genus but only occasionally useful to separate species.

**Figure 11.**
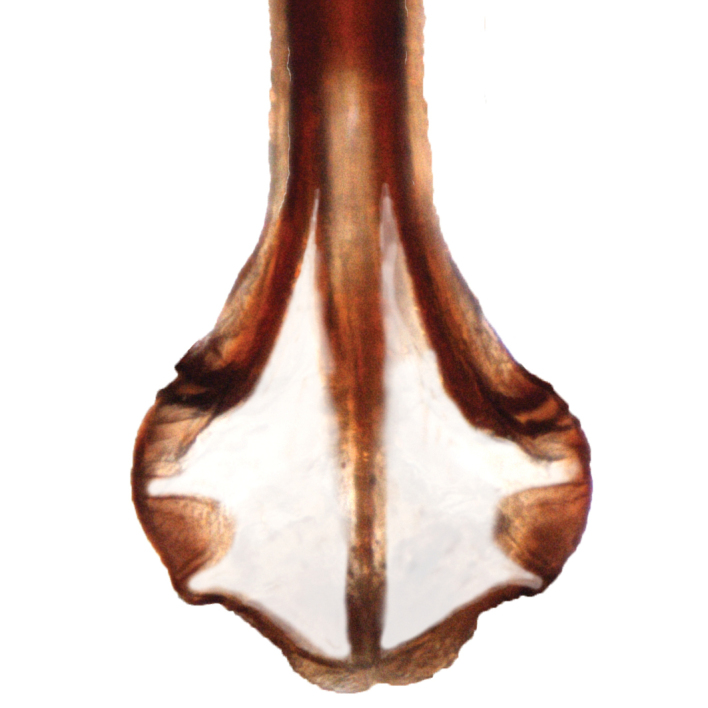
Female, bases of valvulae. Within *Flexamia* these structures provide a means for specific identification of female specimens.

Female. Posterior margin of abdominal sternum VII (Fig. 10) typical of the genus, shallowly concave on either side of the slightly notched and embrowned median convex lobe; ovipositor with bases of first valvulae as in Figure 11.

#### Material examined.

Holotype male, USA: NEW JERSEY: Atlantic Co., Mullica Twp., Batso Fireline Rd., 8.5km SE of Atsion, 27 July 2012 ALHicks, ca. 25 ft asl, 39.6798°N, 74.6705°W. Swept from *Muhlenbergia torreyana*. 6 male and 11 female paratypes, same data.

7 male and 3 female paratypes, NEW JERSEY: Burlington Co., Washington Twp., 10.1 km SE of Atsion, Batso Lk. Rd., 27 July 2012 ALHicks, ca. 20 ft asl, 39.6667°N, 74.6501°W.

Holotype and paratypes in the entomology collection of the University of Colorado Museum of Natural History (UCMC); paratypes in the collection of the United States National Museum of Natural History (USNM).

#### Etymology.

The specific epithet honors an extraordinary mentor, colleague and friend, the late Dr. Robert Whitcomb, who made, among many other accomplishments, major contributions to leafhopper taxonomy and ecology.

#### Diagnosis.

*Flexamia whitcombi* is included in the *serrata* species group by characters of the male genitalia but easily distinguished from the related *Flexamia serrata* and *huroni* by its habitus (dark below and stramineous above). In addition, males can be separated from all other species by the denticulate ventral pygofer process and by the apex of the aedeagus, females by the bases of the valvulae (Fig. 11). Because of its habitus and distribution, it is likely to be mistaken for other eastern species like *Flexamia sandersi* (Osborn) but readily differentiated from this and all other *prairiana* group species by the short, blunt subgenital plates (Fig. 6).

## Discussion

Osborn and Ball (1898) first associated *Flexamia* with *Muhlenbergia* in the description of *Flexamia imputans* in 1898. The recent transfer (Peterson et al. 2010) of *Redfieldia flexuosa* (Thurb.) Vasey to *Muhlenbergia* as *Muhlenbergia ammophila* and this publication brings the total to twelve of 45 *Flexamia* species documented using *Muhlenbergia* as their host. All of the known host species are found in 2 of the 5 subgenera and sections of *Muhlenbergia*—*Pseudosporobolus* and *Muhlenbergia*. (Peterson et al. 2010; Paul Peterson, pers. comm. 2014).

While *Muhlenbergia torreyana* may be characterized as a relatively overlooked part of the eastern US flora, *Flexamia* is, thanks largely to the efforts of the late Bob Whitcomb, a very well-studied non-vector genus of North American Cicadellidae. As of this publication, host-plant associations (at least to genus) are known for 39 of the 45 valid species. Online range maps and photographs (Leafhopper Distribution Maps; Bugguide) exist for a number of species, there is a recent revision (Whitcomb and Hicks 1988), phylogeny (Dietrich et al. 1999), and recent new species description (Bess and Hamilton 1999).

**Figure 12.**
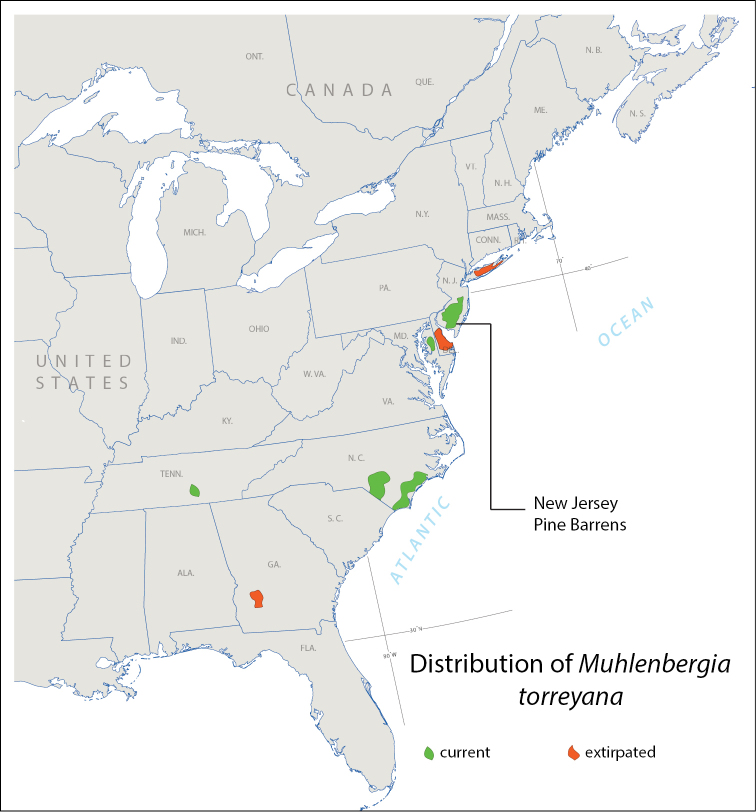
Distribution of *Muhlenbergia torreyana* and location of the Pine Barrens. Modified from data found on the Grass Manual on the Web and BONAP’s North American Plant Atlas.

The relative abundance of *Muhlenbergia torreyana* in the Pine Barrens suggests that it is the epicenter of a host with a very limited distribution (Fig. 12). But to date, as no attempt has been made to look for *Flexamia whitcombi* in Tennessee and the coastal plain of North Carolina where *Muhlenbergia torreyana* occurs, the extent of its range is unclear. The Pine Barrens are already suffering the effects of a warming climate, as evidenced by the recent irruption there of the Southern Pine Beetle (Gillis 2013). Should the effects of climate change or other anthropomorphic pressures cause the local extinction of the host (as has apparently already occurred elsewhere in its range), there will be little opportunity for the survival of this *Flexamia*. But that might be said of most species described today (Dirzo et al. 2014, Kolbert 2014). The description of any new species may serve as a catalyst for additional research, and this will be best accomplished while the species still can be found in nature––something that can no longer taken for granted. To delay the publication of a species description until the time of a genus revision is to deny the pace of change in the natural world in the 21st century and may consign said new species to a future status of “known from a single collection”, or, “presumed extinct, life history unknown”.

### Key to the males of the *serrata* species group

**Table:** 

1	Caudoventral margin of pygofer distinctly thickened, embrowned and terminating ventrally as a denticulate knob; on *Muhlenbergia torreyana*	***whitcombi* sp. n.**
–	Caudoventral margin of pygofer not particularly embrowned or thickened, ventral margin lacking process; on *Muhlenbergia richardsonis*	**2**
2	Apical portion of aedeagus with 5 processes	***huroni***
–	Apical portion of aedeagus with 6 processes	***serrata***

## Supplementary Material

XML Treatment for Flexamia whitcombi
